# Beyond Qualification: A Video-Stimulated Interview Study on What Group Discussion of Audit and Feedback Adds to Professional Development in General Practice

**DOI:** 10.5334/pme.1954

**Published:** 2026-10-12

**Authors:** Dorien van der Winden, Mana Nasori, Marianne Mak-van der Vossen, Nynke van Dijk, Marije van Braak, Jettie Bont, Mechteld Visser

**Affiliations:** 1 PhD student, Amsterdam UMC location University of Amsterdam, Department of General Practice, Meibergdreef 9, Amsterdam, the Netherlands, also affiliated with Amsterdam Public Health, Amsterdam, the Netherlands; 2 Research assistant, Amsterdam UMC location University of Amsterdam, Department of General Practice, Meibergdreef 9, Amsterdam, the Netherlands, also affiliated with Amsterdam Public Health, Amsterdam, the Netherlands; 3 Associate professor, Amsterdam UMC location University of Amsterdam, Department of General Practice, Meibergdreef 9, Amsterdam, the Netherlands, also affiliated with Amsterdam Public Health, Amsterdam, the Netherlands; 4 Professor in Medical Education, Amsterdam UMC location University of Amsterdam, Department of General Practice, Meibergdreef 9, Amsterdam, the Netherlands; 5 Amsterdam University of Applied Sciences, Dean of the Faculty of Health, Sports and Physical Activity and Center of Expertise Urban Vitality, Amsterdam, the Netherlands; 6 Assistant professor at the Department of Language, Literature and Communication, University of Utrecht, Utrecht, the Netherlands; 7 Professor in General Practice, Amsterdam UMC location University of Amsterdam, Department of General Practice, Meibergdreef 9, Amsterdam, the Netherlands, also affiliated with Amsterdam Public Health, Amsterdam, the Netherlands; 8 Senior researcher at Amsterdam UMC, Department of General Practice location University of Amsterdam, Meibergdreef 9, Amsterdam, the Netherlands, also affiliated with Amsterdam Public Health, Amsterdam, the Netherlands

## Abstract

**Introduction:**

Modern healthcare demands continuing professional development (CPD) that bridges learning and practice and motivates health professionals. Current CPD activities often prioritize qualification, while socialization and subjectification purposes are considered vital in other educational contexts and could help to foster dynamic learning. Group discussion of audit and feedback (A&F) is a form of CPD that is used increasingly, and that may fulfill these needs. However, little is known about how this approach creates learning opportunities or what the group dynamic contributes.

**Method:**

In this video-stimulated interview study we explored how a group of experienced general practitioners perceive the value of group discussion of A&F in their professional development, employing a constructivist paradigm. We first inductively analyzed our findings, using Thematic Analysis. A second deductive analysis followed, using Biesta’s concept of the three purposes of education (qualification, socialization and subjectification) as framework.

**Results:**

According to our participants, group discussion of A&F allows for a reflective process that adds to their professional development. The group helps deepen reflection and assists with the forming of individual and collective opinions. The meetings enhanced motivation for both individual and collective learning. Key conditions included a safe learning environment and a high level of enjoyability. The group meetings offered opportunities for all three purposes of education.

**Discussion:**

Group discussion adds value to individual A&F by offering room for socialization and subjectification, as well as classic qualification purposes. It thereby offers a future proof form of CPD that could improve quality of healthcare and stimulate lifelong learning.

## Introduction

The quickly changing landscape of healthcare asks for health professionals that are highly adaptive and continuously work on developing themselves and their practice. Strong strategies for continuing professional development (CPD) are essential to stimulate lifelong learning [1,2]. Yet, existing CPD methods are being critiqued for often being too focused on ‘getting credits’ and for not being properly anchored in practice [3]. This results in lack of engagement with CPD and potentially impairs lifelong learning [2]. We therefore need CPD methods that link learning to practice and encourage engagement. Group discussion of audit and feedback (A&F) combines CPD with quality improvement (QI) of patient care, is strongly rooted in practice and could possibly foresee in this need for a different kind of CPD activity [4].

A&F interventions are traditionally used in QI of care settings [5]. In A&F interventions, health data (audit), is fed back to medical professionals in order to improve patient care [6]. This connects A&F directly to care practice, showing health data that are derived from the delivered care, with the purpose to improve its’ quality. The most recent Cochrane review states that A&F activities have mild to moderate effects [7,8]. Adding a social factor, such as group discussion, seems to enhance the effect [9,10,11]. Currently, A&F is implemented in a CPD context more often, meaning it focuses predominantly on reflection and learning. An example is group discussion of A&F, where health data reports are discussed with a group of peers [4,12]. Since most research on A&F interventions has been done with a QI lens, focusing on outcomes and effect measurement, it is largely unknown how group discussion of A&F works in a CPD setting. It is also unknown why or how a social factor, such as a group, enhances the effects of A&F. To gain more insight in how group discussion of A&F leads to reflection and offers learning opportunities, adopting an educational perspective would be helpful. This approach could identify which key factors influence a successful A&F group discussion and how the group process influences A&F, leading to better design of A&F interventions.

Biesta’s work on the purposes of education offers an interesting education philosophical take on reflection and learning processes. Biesta states that education should not only focus on qualification (transition of knowledge and skills), but also on socialization (understanding and internalizing the values of the profession) and subjectification (relating oneself to the profession and finding your own position) [13,14]. Whereas Biesta’s insights were formerly used mostly in primary and secondary education, they are currently also applied in vocational and (post)graduate training [15]. For example, the new curriculum for general practice in the Netherlands focuses highly on integrating ‘getting qualified’ with socialization and subjectification [16]. In CPD settings though, the focus of both researchers and professionals is still very much on maintaining qualification [2]. This focus stems partly from the design of reregistration systems worldwide, that are based mainly on keeping oneself qualified for the job [2,17,18]. There is little attention for socialization and subjectification, while it seems highly likely that these goal domains also remain relevant after completion of postgraduate training [17,19]. The group aspect of group discussion of A&F raises the question whether socialization and subjectification could play a role in this type of A&F intervention.

In this study we adopted an educational perspective and studied a best practice initiative of group discussion of A&F to explore the following research questions:

1. How does group discussion of A&F offer learning opportunities, according to participants?
2. How do participants believe these meetings add value to their professional development?
3. What factors contribute to establishing a beneficial meeting?

We then explore how these findings relate to Biesta’s purposes of education: qualification, socialization and subjectification, with the purpose of informing future initiatives in practice as well as future research.

## Methods

### Study design and theory

Within a constructivist paradigm, we chose a video stimulated reflective interview format [20]. This suited our objective to explore ideas of participants while sticking closely to what actually happened in the meetings. Our project is two-fold. When designing the project we adopted an inductive set-up, in which we decided not to define learning in advance, but to ask our participants what they found valuable about group discussion of A&F and what they gained from it. This fits Biesta’s ideas on learning: the cognitive process of learning itself is impossible to explicate, while more insight is offered by exploring the presence of valuable moments within an educational activity and how this adds to the development of participants [13]. The second phase of our project was a deductive approach, adopting Biesta’s philosophy on the purposes of education as a conceptual framework.

### Reflexivity

A constructivist paradigm sees knowledge as actively constructed and cocreated as a result of human interactions and relationships. To inform our readers of our own stance, see Table 1 for the authors’ backgrounds. Additionally, all have experience in performing and receiving (medical) education and researching it. The researchers are affiliated with a large general practice research and training institute. The overarching research project came to exist through a request posited by a group of local general practitioners (GPs), asking for a scientific framework for their CPD activities. This stimulated us to keep our research aim closely connected to practice, while also furthering theory. DW’s own experience in general practice offered the opportunity to stick closely to the context. However, it may have also created a narrower mindset, which is why adding researchers with different backgrounds was important. We maintained a reflexive stance throughout the research process by frequently discussing our positionality and critically questioning each other on its implications.

**Table 1:** Background of the authors.


AUTHOR	BACKGROUND

DW	GP in final stages of training PhD student in medical education

MN	Health policy PhD student in patient participation

MM	GP Medical educator/Assistant Professor in HPE

ND	Professor in medical education Dean of HPE faculty

MB	Assistant Professor in communication studies Language specialist

JB	GP Professor in general practice

MV	Senior researcher in Medical Education Cognitive psychologist & Quality expert


### Context

We conducted our study within a general practice setting in the Netherlands. Dutch GPs need to reregister every five years [21]. For reregistration, GPs must dedicate at least 10 hours per five years to peer-review CPD activities, such as group discussion of A&F. To facilitate peer-review activities, GPs participate in voluntarily formed groups. Group dynamics vary significantly among different groups, as some groups are collaborating intensively in several respects, while other groups only come together for the obligatory hours of peer-review.

### Participants and set-up of meetings

We purposively recruited an established group of collaborating GPs who had been participating satisfyingly in group discussion of A&F for several years. Studying this “best practice” group fitted our research questions: it allowed us to broaden our understanding of how group discussion can be valuable and offers insight in what factors are pivotal for this. This group, consisting of 12 GPs, developed their own format for their meetings over time. The subject of the meeting is determined by the members of the group. The subjects that were discussed in the meetings we observed were ‘Menopausal symptoms and their treatment’ and ‘Osteoporosis in the GP practice’. A short questionnaire on the subject is sent out beforehand, covering knowledge items based on the national guidelines on the subjects and self-report on how well a subject is mastered (e.g. ‘How well do you believe you master this subject on a scale from 1 to 5?’). This questionnaire is made by the member of the group that chairs the meeting, a role that rotates every meeting. The meeting contains a presentation by the chairing group member on the subject, followed by a discussion of A&F reports on practice level. In some meetings they are joined by an external expert on the discussed subject (a specialised GP or medical specialist). The meeting ends with stating points of improvement by each member, which they revisit after a year, to map progress. For these meetings participants can claim accreditation points, depending on the time they spent on the meetings (1 accreditation point for each hour), but not specifically linked to outcome improvement. These accreditation points are appointed for participating in peer-review CPD activities, of which A&F is one of the options. Doing a form of peer-review CPD is mandatory, but taking part in these A&F meetings is voluntary.

### Data collection and interviewing

Data collection and analysis was performed iteratively. We observed and video recorded two regular A&F group meetings. During the meetings we tried to be considerate of our presence, influencing the process, by being as much fly on the wall as possible. After the first meeting the video material was viewed by two individual researchers (DW and MV) with the purpose of identifying key learning opportunities in the meeting. Videos were then watched together by DW and MV, reaching consensus on which opportunities to discuss within the interviews that would follow. When necessary, a third researcher (ND) was consulted. After this, we conducted a round of individual video-stimulated interviews. Participants were selected based on their availability, preferably within a three-week window of the meeting. Semi-structured interviews were performed by DW and MV in a location chosen by the participant. At the beginning and the end of the interview, participants were asked if they recalled moments they wanted to review. Because all participants indicated that they would not have time to review the footage themselves, we used video fragments that were selected by the researchers. For each participant, we used a basic topic list. This topic list was constructed through discussion in the research team, allowing for some general questions on the participant and their work in the practice, their general ideas on group discussion of A&F, then shifting to the video fragments. The latter part was constructed by the research team, for each individual interview, based on the video material. The questions that were asked followed the same principle of asking the participant what their reflection was on the fragment in an open manner. These topic lists structured the interview while also offering space for questions that arose. [See additional materials 1 for a general example] [22] This topic list was developed through discussion within the research team. The interviews were audio-recorded and transcribed verbatim. For the second meeting the same format applied.

### Analysis

We used Thematic analysis to analyse transcripts of the interviews [23]. Its flexible and overarching approach suited our broad research objectives. We used MAXQDA software [24]. Analysis was done by two researchers independently (DW and MN), consulting each other after each interview and iteratively building on a shared coding tree. In this first inductive analysis, open coding was used. MV was consulted after every two interviews. Through the collaborative process of discussion, a shared understanding was reached on a final coding tree. [see additional materials 2] After analysing the second round of interviews, we evaluated findings with the whole research team and concluded that our data was sufficiently rich to answer our research questions.

After inductively analysing all transcripts, the research team recognised that the findings fitted Biesta’s work on the purposes of education [13,14]. For example, parts that we recognised as ‘deep reflection’ in our inductive analysis, could also be read as ‘socialization’ and ‘subjectification processes’, as described by Biesta. Even though we initially chose an inductive setup, after discussion we considered it paramount to examine this link more explicitly. Therefore DW and MM went back to the raw data for a second, deductive round of coding. Here, we used Biesta’s three purposes as a priori codes, forming our framework. Where we believed there was overlap in the purposes we initially labelled all applicable purposes to the data. Then, in order to decide upon the most applicable purpose in case of overlap, we we discussed these parts more thoroughly, weighing again the context in which statements were made, and trying to do justice to Biesta’s complex and nuanced philosophy [25].

Subsequently, after discussing the results with two additional researchers (MN and MV), the final deductive coding tree was constructed. [see additional materials 3] A shared agreement on the final results was reached within the full research team.

We believe that our deductive analysis fortified our inductive findings, explaining them more clearly. This aligns with established perspectives on the inherently iterative and reflexive process of qualitative research, which does not always follow a chronological order [26]. It also fits existing ideas on how inductive and deductive methodologies could enhance one another and serve the data together [27,28].

### Ethical consideration

The Medical Ethical Board of Amsterdam UMC regarded the study protocol not to be subject to the Medical Research Involving Human Subjects Act. Consequently, they provided a waiver (numbered A1 19.333). All data obtained within this study was processed and stored according to the General Data Protection Regulation and according to the Amsterdam UMC Clinical Research Unit procedures. In addition to an information meeting on the study’s purpose and privacy concerns, all eligible participants were sent an information letter. All participants individually gave informed consent for observation and video recording of the group meetings. Participants who agreed to observations and video recordings of the meetings were allowed to opt out of participating in the interviews.

## Results

Video recordings were made at two A&F group discussion meetings. Set-up of the meetings was similar, although the meetings differed slightly since in the first meeting the knowledge background was shared by the chairing member of the group, while in the second meeting a content-related expert was present, being an external GP with extensive knowledge on the discussed subject. This external GP is not a member of the peer-review group. Eight interviews were held with seven participants. The moderator of the group was interviewed twice, i.e. after each meeting. See Table 2 for participant characteristics.

**Table 2:** Participant characteristics.


CHARACTERISTIC	DETAILS

Number of participants	7

Profession	6 GPs; 1 resident

Type of practice	4 duo practices; 2 solo practices; 1 group practice

Years of experience as a GP	2–25 years; 3^rd^ year resident

Time participating in the group	First time – 5 years


In the following paragraphs we first describe our inductive findings, answering our research questions. Subsequently, we depict the deductive results, indicating how qualification, socialization and subjectification are present in our findings.

### Deep reflection

Participants explained that the group aspect of the meetings was particularly valuable. The A&F reports were said to connect the meeting to daily practice and were used to initiate reflection. Participants explained that, when confronted with A&F that stands out from the rest in a negative sense, others helped them to reflect by asking questions and thinking along, prompting questions such as: why is my A&F so different from the others? Do I organize my practice differently? Do I have different values or a different idea on what is proper care? This reflection led to important insights, aiding in identifying potential issues, areas for improvement, or aspects that required further investigation. The group was considered assistive to establish *a more meaningful level of reflection*, harder to reach individually. (Table 3, quote 1)

**Table 3:** Quotes 1 and 2: Deepening of reflection.


*1*.	*‘On the other hand, there are topics where you can have strong opinions, and as a group, you might be able to question each other, so that you get to a deeper layer of “Oh, what are actually my assumptions about this, and what effect does that have?”’*	*Participant 1*

*2*.	*“Yes, on your own, you look at things in a certain way. I do my medicine the way I always do it, so I also think I look at those numbers (i.e. the A&F report) the way I would usually look at them. But when others say something about it, you do hear things from outside your normal way of working.”*	*Participant 3*


Also, participants described that even if they were not the one actively reflecting, it was still helpful to hear others talk about the subject and reflect on their practice. This offered perspective on participants own situation or functioning. It also contributed to forming new ideas and opinions of their own. Oppositely, when doing relatively well compared to the others, this enhanced participants confidence and even resulted in more job satisfaction. (Table 3, quote 2)

### Individual as well as collective learning

Also, the group process was described to be pivotal for *forming both individual and collective opinions*, which happened by discussing the subject and hearing ideas and opinions of others. Subjects that especially benefitted from group discussion, were those for which there is limited evidence, or on which professional guidelines leave room for interpretation: participants felt the need to discuss these subjects with their colleagues and *form a collective opinion* on how to tackle these subjects. Since in daily practice they often work solo, these group meetings filled a gap, in this respect. The phenomenon of collective learning brought joy and resulted in motivation to perform their profession and to keep learning. (Table 4, quote 3)

**Table 4:** Quote 3: Inducing individual and collective learning.


*3*.	*“So, by grappling with the material, by discussing or questioning each other, I understand it… Yes, or ‘understand it,’ I do, but it also makes me remember it better. It also offers me pleasure to go do something with it again.”*	*Participant 7*


### Collective action

Another effect of the group meetings that was described, is that participants identify common problems in the meetings, often caused by working in the same setting. These could be solved by *collective action*. A mentioned example was problems with referral of patients to the local hospital. Participants said the meeting empowered them to take collective action, while they would not have felt able to do so individually. (Table 5, quote 4)

**Table 5:** Quote 4: Collective action.


*4*.	*“…but then a second goal of such a community health group is democratization. If something is felt in the field, it must be communicated to hierarchies above this group, so that it is actually addressed there. … So now you need to try to generate enough volume for the issue and then push it forcefully to the right place. Yes, that then becomes a bit of a political piece. That can also be the outcome of the* [group discussion of AF] *session. Yes.”*	*Participant 1*


### Mapping our findings to Biesta’s concepts

In our deductive analysis phase, we mapped our findings to Biesta’s concepts. All three functions of education could be recognized in our participants descriptions of learning from A&F group meetings. *Qualification* was present as looking at the numbers and seeing how well the participant was performing (as a means of assessment) and in the information/ knowledge sharing part of the meetings. Here either the chairing member of the group, or, if present, the subject expert, explained the guideline/status quo on the subject of each meeting. This resulted in feeling up-to-date and adequately informed on the discussed subject, according to our participants. (Table 6, quote 5)

**Table 6:** Quotes 5–11: Qualification, Socialization and Subjectification.


*5*.	*‘Most of them are really experienced doctors, and they’ve been working in a certain way for twenty years, but they also have an exceptionally good clinical eye and a lot of experience. Look, working the same way for twenty years isn’t good, of course. You must keep up with the times, and you need to be flexible. That’s definitely what’s being discussed properly here.’*	*Participant 3*

*6*.	*‘*It’s really about searching together for “how do we do this now?” … It’s definitely about searching together for “what motivates us to do it the way we’re doing it?”’	*Participant 2*

*7*.	*‘Of course, there are topics where there is much more of a norm. Sometimes, it’s like everyone is deviating from the norm, like with oxycodone, and you can still learn from each other in how you deal with it and the solutions you’re going to look for, talking about it together, like “who would know more about this, who could we possibly refer to, how do we prevent this exactly, what’s our first choice pain relief,” giving that some structure. Then you’re searching together, and you have consensus on the norm…’*	*Participant 1*

*8*.	*‘What do you do in that consultation room? You must think about that. And then you need to think further; the next step is “how do you want to do it?”’*	*Participant 2*

*9*.	*‘I practise medicine the way I always do, so I suppose I look at those numbers the way I normally would. But when others comment on them, you start hearing things from outside your usual way of working. It pulls you a bit out of your comfort zone — and that’s actually a good thing.’*	*Participant 3*

*10*.	*‘You’re expected to respond right away and have an opinion, even while you’re still thinking, ‘What’s actually going on here, what am I supposed to do with this?’ What I do know is that I want to pay more attention, be more aware of it, really listen to these complaints. But at the same time I’m also thinking, ‘Hang on, wait a second.’ I don’t see it as something black-and-white. That’s something I try to bring into the conversation too. Is it really that wrong?’*	*Participant 3*

*11*.	*‘Well, it does make you think. ‘How come I pay so little attention to these menopausal symptoms?’ And then I wonder, ‘Is that something about me?’ I’ve always been the type to say, ‘Come on, don’t make a fuss.’ And I think I carry that attitude into my consultations as well. Which is fine, in a way — some things aren’t good or bad, they’re just different. But it does make me wonder, ‘Am I maybe short-changing some women because of that?’ And that really gets you thinking.’*	*Participant 3*


*Socialization* was recognized in participants description of what group discussion led to: when discussing subjects for which there is little evidence, or where guidelines leave room for interpretation, this resulted in a shared construction of values, norms and ideas on what they believe good GP care is. This was considered one of the main functions of the meetings: by discussing subjects, a collective concept formed around the discussed subject.(Table 6, quote 6 and 7)

*Subjectification* was clearly identified as both an objective for attending the meetings and as a result of the meeting. Through the process of discussion and forming opinions as a group, participants discovered their own position in relation to the discussed subject. This was often prolonged after the meeting: back in their practices this thinking process continued, for example through conversations with their direct colleagues. Additionally, this resulted in relating themselves to their profession: what kind of doctor do I want to be? What do I find important? What do I believe is the right care, and how does this become visible in my daily doctoring? (Table 6, quote 8, 9, 10, 11)

### Preconditions for a beneficial meeting

The *safety of the learning environment* was considered essential because it created space for vulnerability, which our participants deemed necessary for reflection and learning. Participants described this as a ‘there is no right or wrong’ atmosphere. This *safe learning environment* was actively created by key figures, such as the moderator or other experienced GPs in the group, making themselves vulnerable and openly showing their insecurities. This encouraged other group members also feeling safe to share their own experiences. At the same time, the presence of these experienced role models could be intimidating for some participants: putting them on a pedestal could also impair the willingness of participants to share their insecurities. (Table 7, quote 12 and 13)

**Table 7:** Quotes 12 and 13: Safety of the learning environment.


*12*.	*‘At some point, there were names of practices involved . Yes, then you have no choice but to expose yourself. But even then, there’s an atmosphere of “there’s no right or wrong”.’*	*Participant 5*

*13*.	*‘You asked: ‘What’s it like, being in such a group?’ The fact that I don’t mind Ma. asking me that… It’s also a very safe group. That absolutely helps.’*	*Participant 2*


*Enjoyment* was considered crucial for the meetings to continue. Participants described enjoying the meetings, which was a key reason to join them and to keep showing up. The moderator considered making the meetings enjoyable a large part of her assignment. This also influenced her behavior as moderator: ensuring enough laughs and adding jokes when things got heated or someone was called out. This was connected to the non-compulsory nature of the meetings but was also considered helpful for learning. (Table 8, quote 14)

**Table 8:** Quote 13: Enjoyment.


*14*.	*‘Everyone should enjoy it a bit and has to want to come, because I have no other leverage, so I just have to make it fun.’*	*Participant 1*


### Purposes could be conflicting

The meetings covered all three purposes, although among the participants some conflicting wishes were present. Participants that favored qualification as the main purpose, expressed a strong wish for an expert to be present. Opposingly, a participant that recognized the priority of subjectification, said that the presence of an expert can impair discussion among the group, overshadowing the group process. Since the expert is there to explain what is right and what is wrong, this allowed the group less room for discussion and to explore their own ideas. (Table 9, quotes 15–17)

**Table 9:** Conflicting purposes.


*15*.	*‘Well, the woman who knew the most about it came across as very definite about a lot of things she said. And that… yeah, I would’ve liked a bit more nuance there. But at the same time, it does stick with you.’*	*Participant 7*

*16*.	*“Well, I can tell you my goal — and I’ve already told you that… I just automatically assume that everyone has the same goal. But yeah, you’re making me realise that there are fifteen people sitting at that table, each with their own agenda.”*	*Participant 4*

*17*.	*‘…and that’s also generally the flaw of bringing in an expert, because it’s much easier to just pick up those pieces of knowledge again than to reflect.’*	*Participant 1*


## Discussion

In this article we set out to understand how participants believe group discussion of A&F offers learning opportunities and how these meetings add value to professional development of GPs. We examined which factors contributed to establishing a beneficial meeting and explored how our findings relate to the three purposes of education, i.e. qualification, socialization and subjectification. Our results show that our participants believe learning opportunities are created in twofold: the A&F reports serve as the start of a reflective process that is shaped by the group discussion process. The group offers learning opportunities by helping to *deepen reflection* and with forming *individual and collective opinions*, especially on subjects where less evidence is available. The meetings stimulated motivation for individual and collective learning. Offering room for reflection and discussion was believed to be the main addition to professional development of, and according to, participants. Our search for beneficial factors led us to two key conditions to make these meetings successful: the presence of a *safe learning environment* and a high level of *enjoyability*. The group meetings offered opportunities for all three domains of purpose of education: *qualification*, *socialization* and *subjectification*.

Below we discuss these results by circling through three important findings: how the group added value, the stimulation of collaboration and having room for socialization and subjectification purposes, as well as the more traditional qualification that is often focused on in traditional A&F.

### Learning opportunities: The added value of the group

As we set out to broaden our understanding of what the group adds, when studying A&F, we found that the added value is most present within the process of discussing the A&F together: learning opportunities occur within and through discussion (Figure 1). This resonates with the broader educational literature on social learning, starting with Bandura’s theories, published in the 1970’s [29]. This theory describes learning as an inherently social process: learning begins with looking at peers. These principles are widely used in primary, secondary and undergraduate education. Our exploratory findings within this established group of collaborating GPs that had been satisfyingly participating in A&F group discussion before, suggest that it is also relevant in this CPD setting, even though traditionally CPD is often an individualistic undertaking. Our results indicate that the aspect of learning together with colleagues is especially appreciated and even motivates participants to keep learning, both individually and collectively. This insight within the CPD context may be an important one: the focus of CPD would probably profit, from a shift from the individual to the collective and make more use of group interventions such as group discussion of A&F.

**Figure 1:**
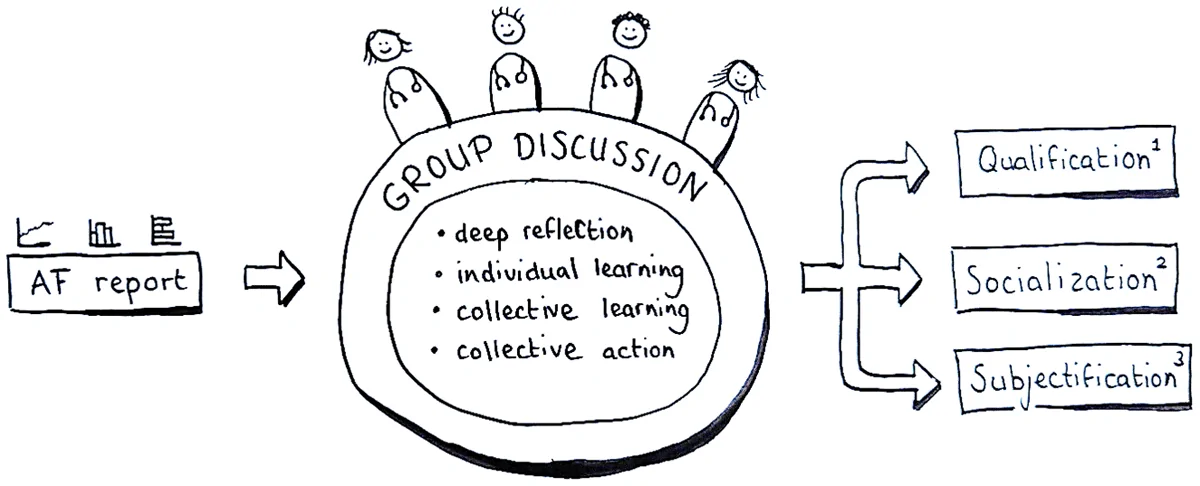
Visual summary of results; 1. Qualification, understood as: transition of knowledge and skills; 2. Socialization, understood as: understanding and internalizing the values of the profession; 3. Subjectification, understood as: relating oneself to the profession and finding your own position.

### Learning opportunities: Stimulating collaboration

Our results show that these group meetings created the opportunity for collective action. This suggests that using more group based CPD activities could also stimulate collaboration and understanding between professionals. Both are pivotal for modern high-level healthcare [30,31]. For general practice this stimulation of collaboration is especially relevant, as daily practice of GPs is highly based around the individual doctor. In the meetings our participants found space to come together, learn together and to gain a better understanding of their own work and that of others.

### Adding to professional development: Beyond qualification

Broadening our view on CPD through incorporating socialization and subjectification, into the more traditionally qualification focused CPD setting, may help to shape CPD towards a more future prove form. Our participants indicated that one of the much appreciated gains of group discussion of A&F was the process of coming together, forming collective insights and understanding their individual stance on the discussed subject. Group discussion of A&F really added to their professional development, as there is not much time and space for this in regular practice, they pointed out. Mapping our findings to Biesta’s purposes of education, resulted in deepened understanding of our findings:Within the moments of ‘deep reflection’, we recognized processes of socialization and subjectification. Whereas, participants feel that qualification should still be strongly represented, it also seems that socialization and subjectification will enrich the broader curriculum of lifelong learning. Even though all three purposes are not traditionally used for a CPD setting, our study indicates that in group discussion of A&F combining all three is a strength. It fits recent findings that a broader conceptualization of lifelong learning and CPD is necessary, focusing not only on qualification, but additionally offering space for socialization and subjectification [2,19]. In our study we showcased this by providing a real-life best practice example of how combining the three purposes helps professionals in their development. We thereby add to the evidence that seeing CPD in a broader context by acknowledging all three functions, could possibly help professionals to better navigate the changing landscape of healthcare [2,17,19].

Notably, in the CPD setting, we may need to consider a broader definition of socialization, than traditionally used in formal undergraduate and graduate training settings. In the latter, socialization is often defined as ‘making the norms and values of the group your own’. This process is now acknowledged as an important part of undergraduate and graduate training. [refs] Our results showed that, in this CPD setting, especially lesser researched subjects and subjects where guidelines leave room for interpretation, invoke discussion. This discussion resulted in forming an opinion as a group and deciding upon norms and values (‘what do we believe good/proper care is?’). We argue that this is what socialization could look like in a CPD setting: not only conforming to existing norms and values of the profession, but also forming new ones.

This broaderdefinition fits Brown and Finns concept of ‘post-functionalist’ socialization, and reads as a more fluid definition of socialization: one where the norms and values are only partly set, and can still be shaped [32]. This definition seems to fit the conditions in which health professionals work and learn more accurate than the smaller definition often used in graduate and undergraduate training, again helping them to better navigate in the changing landscape of healthcare.

### Preconditions for a beneficial meeting and conflicting purposes

Applying Biesta’s insights poses the question of how to interpret the importance of a safe learning climate, and of enjoyment, in this light. The need for a safe learning environment is widely spread throughout pedagogical and educational literature, also fitting Biesta’s description of the need for ‘objective spaces’ to find answers [13]. This objective space, where learners feel safe to explore the world, to learn by discovering and making mistakes, is vital to learning, or rather, to facilitate qualification, socialization and subjectification. In Biesta’s theory, the teacher is an important player in providing this space. In group discussion of A&F, we believe that the moderator in collaboration with the group can provide this ‘objective space’ by ensuring vulnerability.

In these meetings, enjoyment contributed to a more engaging and productive learning environment. However, focusing on enjoyment poses a danger: if all friction is avoided, learning could be impaired. According to Biesta this is exactly one of the dangers of modern education: continuously trying to make learning fun, results in missing important learning opportunities [14]. Learning cannot always be pleasant: sometimes learning is hard, or even unpleasant. Our data show that the moderator plays a pivotal role in both the safety of the learning environment and properly balancing enjoyment. We therefore recommend to carefully select the moderator, who is preferably a key figure from within the group, leading firmly but also showing and allowing vulnerability in a role model position.

One of the roles of this moderator may be to navigate conflicting purposes. Our data showed that, while some people are in the meetings in order to boost their knowledge (qualification), others wish to question their premises and reflect upon their practice in a more profound way (deep reflection/subjectification) or people may want to form collective opinions and/or action on one of their pain points in their joined context (socialization/collective action). According to Biesta, navigating all three purposes is believed to be one of the key tasks of the teacher. For this, there is no set formula, but this requires ‘phronèsis’, or teacher judgement. Here, the teacher uses their skills to consider, in each moment, how to balance the purposes. The moderator in A&F meetings, is not a classic teacher, but, as it seems, does need to navigate all three (sometimes conflicting) purposes. This demanding task encourages the idea that the moderator must be someone with ‘weight’ in the group, that caries their experience [14].

### Strengths and limitations

In this study we furthered our understanding of A&F group discussion by looking at it through an educational lens. This educational lens provided us with insights in the process of the meetings, looking at how it offers learning opportunities and what it actually adds to professional development. This perspective into the process is one that the QI lens often misses, but that offers essential knowledge to successfully design interventions that will make a difference. To our knowledge, our study is the first to use this focus from an educational perspective. Through making the process more insightful, we show that this is a valuable perspective that contributes to the understanding of A&F. Additionally, this study is the first to gain an understanding of the participants’ perspectives of A&F group discussion by using a video-stimulated interviewing setup. This makes the insights gained from the interviews strongly connected to what actually happens during the meetings. Combining our inductive findings with an additional deductive analysis, in which we utilized Biesta’s purposes of education as a framework, fortifies our findings, explaining their meaning and offering opportunities for further research. Even so, our study is one of modest proportions: it is exploratory and participants are all part of only one CPD group. The well-established group seemed to offer a safe learning climate for the participants, which resulted in opportunities for learning to take place. Such a best practice example, shows ‘what is possible’, but may not be realistic in all settings and collaborations. Therefore our findings require further research to see whether they resonate with various groups in various contexts and further exploration on how to establish a group that can elicit these opportunities for learning. The video stimulated set-up means participants were filmed during their meeting. It is possible that filming of participants may have altered their behaviour. This is an effect that is part of the video stimulated set-up. We believe that the benefits of the video-stimulated setup balances this possible bias. In video stimulated interviewing studies, it is preferred to allow for the participant to select their own fragments. Due to practical reasons (lack of time of our participants), in our study the research team selected the video fragments that were discussed in the interviews. This may have influenced our findings: the purpose of letting participants select their own fragments allows them to access their tacit knowledge by choosing those moments that instinctively feel valuable to them [20]. The fact that we selected the fragments, rather than participants themselves, may have caused more metacognitive, and less tacit, knowledge to rise from these data. Finally, studying a best practice group means that we interviewed people who are experienced with, and mostly enthusiastic about, the format. This fitted our purpose of broadening our understanding of what we could gain by adding group discussion to A&F interventions, but it is good to keep in mind that it does not shine light on potential problems and risks.

### Implications for design, practice and future research

Understanding that group discussion of A&F is not just about qualification, but also offers space for socialization and subjectification, can help in designing, evaluating and researching this type of intervention more effectively. All three purposes in mind, we understand better when and how to use group discussion of A&F. As we see in our results that purposes can coexist, but also be conflicting, this asks for moderators that acknowledge different purposes and realize that all purposes need to receive attention. For practical implications for the design of A&F group discussion interventions, see Box 1. As for implications on future research: when researching this type of group intervention, we need to rethink our setups: we will need to map outcomes and effects beyond just qualification, and closely examine the process, as we get a better understanding of what health professionals can gain from it. Additionally, gaining insight in how group dynamics make or break these meetings, could further help our understanding of what makes group discussion of A&F beneficial. Examining potential risks and problems regarding group discussion of A&F would shed a better light on barriers. The latter is an essential part of properly implementing the meetings in practice.

**Box 1 Praticical implications when designing A&F group discussion**

**Practical implications when designing A&F group discussion:**

- Consider the purpose of the meetings: solely qualification or more?
- Be aware that different participants may have different purposes for the meeting and that these purposes can be conflicting.
- Be aware of the pivotal role of the moderator: in navigating conflicting purposes, ensuring enjoyability, while allowing for friction.
- Allow for the group discussion to take up a large part of your intervention: this is where the learning process is shaped.
- Create space forsocialization and subjectification; this is done by trusting the group process and allowing time for informal discussion.
- Be aware of how safe the learning environment feels: examine which factors ensure and threaten it.
- Include enjoyment as an essential element: it motivates and inspires.

## Conclusion

Concluding, group discussion of A&F adds value to professional development in general practice by deepening reflection and allowing for individual as well as collective learning and action. Group discussion adds value to individual A&F interventions by offering room for socialization and subjectification, as well as classic qualification purposes. The group process motivates and stimulates collaboration. It thereby offers a future proof form of CPD that could improve quality of healthcare and stimulate lifelong learning.

## Additional Files

The additional files for this article can be found as follows:

10.5334/pme.1954.s1 Additional materials 1. Topic List – English.

10.5334/pme.1954.s2 Additional materials 2. Codebook Inductive Analysis.

10.5334/pme.1954.s3 Additional materials 3. Codebook Deductive Analysis.

## Data Availability

Due to privacy concerns our original data are available through the corresponding author upon request.
