# Supplementary material for: Beyond Qualification: A Video-Stimulated Interview Study on What Group Discussion of Audit and Feedback Adds to Professional Development in General Practice
Source: Perspect Med Educ. 2026 Oct 12;15(1):1073–84. doi: 10.5334/pme.1954 (PMC13674528; doi:10.5334/pme.1954)
Supplement: Supplementary file 2 [file pme-15-1-1954-s2.pdf]

Codebook Inductive Analysis

Deze groep	0
Doel van bijeenkomst	0
Ervaring op doen en sociale ontmoeting	1
Spiegelen 'wat voor mens ben ik op dat gebied'	1
Dualiteit over doelen	0
Dualiteit binnen 1 persoon	0
Gezamenlijk doel	0
Het doel maakt niet uit	1
Bespreken wat bij de visie van de wijkgroep past	1
Alle andere doelen zijn daaraan ondergeschikt	1
Doel is niet om antwoorden te krijgen op vragen	1
praktijkvariatie zo klein mogelijk krijgen (soms)	1
Gezamenlijk doel wordt niet besproken	3
Eigen doel	0
Leervraag beantwoord willen krijgen	1
Verwachting: wat kan ik halen en kom ik brengen	1
Eigen ervaring delen	1
Samen op zoek gaan naar "hoe doen we dit nu?"	1
Doel is reflecteren op eigen gedrag	1
Doel is niet expertise delen	1

Leren	0
van elkaars leerpunten	0
een soort groepsdruk om leerpunten te bedenken	1
Leerpunten van elkaar gebruiken	3
Leerpunten kunnen stimulerend voor de groep werken	2
Het blijft net wat beter hangen zo'n lijst met leerpunten	1
Blinde vlekken	1
Leren van elkaar	0
door te discussieren/elkaar ondervragen	1
hierdoor onthoud je het beter en leer je	1
Erachter komen wat jezelf aan een ander te brengen hebt	1
Profiteren van anderen, veel dingen opschrijven	1
De weerstanden als mens/dokter komen naar voren in zo'n groep	1
Je basishouding wisselt	2
m.b.v. expert	0
De vraag werd niet goed begrepen door expert	1
Kritischer dan eerst of het klopt wat de expert zegt	1
Expert had meer kunnen uitleggen over interpretatie van VFA	2
De expert is soms stellig waardoor het blijft hangen	3
Behoefte aan meer nuancering	1
Vakinhoudelijke sturing van iemand die boven de stof staat	2
expert als huisarts	0

Expert geeft weinig ruimte voor discusse bij take home message	1
In korte tijd (te)veel informatie zenden	2
Expert als huisarts geeft goeie home messages mee	5
Vervelend dat expert zonder toestemming boodschap deelt	1
expert 2e lijnszorg	0
Mening vd expert kun je goed toetsen aan wat de groep vindt	1
Leren wat je interdisciplinair voor elkaar kunt betekenen	1
er achter komen wat de blinde vlek is	2
Voorschrijven van vitamine d minder voorschrijven	2
Zou een meerwaarde zijn	1
Die vakinhoudelijke sturing geeft bij verschillen in handelen	1
Miste iemand die boven de stof staat	2
Van expert horen wat de voor- en nadelen zijn, huidige kennis	1
Efficient om je verhaal aan meerdere mensen te vertellen	1
Die meer kan vertellen over wat er bekend is	1
die kan je vertellen of je het goed doet of niet	1
Transmurale afspraak	1
d.m.v. casusbesprekingen	0
Je leert niet van casuïstiek	0
Eén specifieke casus zegt niets hoe je het in het algemeen doet	4
Adhv cijfers reflecteren op eigen handelen mbv associatief denk	1
Huisartsen vinden het prettig om in plaatjes te denken	2

Casuïstiek is niet zo interessant/relevant	5
Casuïstiek bespreken voelt als expertise delen	1
d.m.v. zelfgerapporteerd gedrag	1
Zelfgerapporteerd gedrag is sensitiever dan de cijfers	1
Helpt om toch wat in de cijfers te zien	1
Veel signaleren, maar weinig coderen	1
Veel signaleren, maar weinig voorschrijven	1
d.m.v. persoonlijke ervaring	0
Je leert anders als je niet direct mee te maken hebt gehad	1
Leerzaam om ervaringen uit het veld te horen	1
Eigen praktijkervaring ontbreekt	2
Zelf geen ervaring met dit onderwerp	1
Lastig om een gefundeerde mening erover te hebben	1
Kan alleen op metaniveau hier iets over zeggen	1
Uittunen als het prakti is en je zelf er geen ervaring mee hebt	1
Je herkent de praktische dingen dan niet	1
Gaat over jezelf, maar heeft weerslag op hoe gesprek in gaat	1
Uitleggen hoe je er in staat	2
d.m.v. cijfers	0
Heb je dingen weer even helder: hoe doe je het?	1
Het doet altijd iets voor je praktijk	1
Het nut spiegelinfo is dat huisartsen alleen id spreekkamer zit	1

Je wordt geconfronteerd met hoe je het echt doet id praktijk	4
confrontatie is niet altijd leuk, maar doet iets voor je prakti	1
Je hebt een beter beeld als je de patiënten id praktijk kent	2
Het zet je aan het denken en geeft stof voor daarna	1
Geeft een soort overzicht	1
Kan zelfvertrouwen verhogen, daardoor ook werkplezier	1
Cijfers verdwijen naar de achtergrond	1
discussie over cijfers	0
Geen toegevoegde waarde, zie mezelf niet id cijfers terug	1
door groep dingen buiten je normale manier van werken meekrijge	2
Alerter op je eigen blinde vlekken	1
Je gaat meer nadenken over hoe je handelt	1
Niet open genoeg over de verklaringen gediscussieerd	1
Discussie volledig door elkaar heen	0
De discussie laten gaan en zelf uitvogelen hoe je het doet	2
als cijfers niet kloppen/niet specifiek zijn	0
Helpt als nog goed om te zien wat jouw praktijk verwerkt	2
Maakt niet uit, bijeenkomst blijft interessant	1
roept frustraties op over het overkoepelende logistiek/HIS	1
Nadenken over waarom het dan niet klopt	2
Nadneken of het door ICPC coderen komt	2
Maakt niet dat je er niks mee wil doen	2

Je doet er niks mee door de hecktiek van de dag	1
Daardoor haakt iedereen af	1
discussie gaat dan over de cijfers die niet kloppen	1
Weinig gefundeerd	1
De cijfers waren weinig tastbaar	3
Niet aangesproken voelen door de cijfers	2
Minder geneigd om te leren als gedrag niet weerspiegeld wordt	1
Niet weten of de cijfers dan ook kloppen	1
Parameter is te ver van wat er in de spreekkamer gebeurd	1
Als de cijfers niet kloppen dan leer je niet	2
als cijfers specifiek zijn	0
Cijfers moeten ramvast staan om gemekker af te vangen	1
Op deze manier kun je het echt over gedrag hebben	1
Je kunt er niet onderuit draaien als de cijfers specifiek zijn	1
Goed bij de cijfers stilstaan wat ze betekenen	1
Moeilijk te volgen, tijd nodig om de cijfers te begrijpen	0
Cijfers zie je in de groep voor het eerst	1
Je zou eigenlijk willen leren wat er achter die cijfers zit	1
Niet meteen naar verklaringen zoeken	1
HIS uitkammen is niet spiegelen	1
De leerdoelen komen niet van de cijfers	1
Door declaratiecijfers blinde vlek ontdekken	1

Soort praktijk maakt niet uit voor hoe ze leren	1
Door verkeerde parameter alert worden gemaakt op blinde vlek	1
Niet interessant	0
Gemiddelde	0
Het voelt niet vervelend om bij de rare uitschieters te zitten	2
Je eigen cijfers vergelijken met het gemiddelde	1
Van het gemiddelde afwijken is niet goed	1
Verklaring hebben waarom je het anders doet	1
Moeten verantwoorden waarom je niet bij het gemiddelde hoort	1
Het landelijke gemiddelde is goed	1
Je eigen expertise ontkennen en bij de hoop willen horen	1
Afwijken tov andere praktijken	0
Soms wel vervelend, maar je bent hier om te leren	1
Hoe anderen het doen kan eigen zelfvertrouwen verhogen	1
Er heerst een 'geen goed of fout' sfeer	1
Cijfers waren nodig om te beseffen dat je afwijkt	1
Individueel afwijken zorg voor besef	1
d.m.v. meningsvorming	3
Gaat over jezelf, maar heeft weerslag op hoe je gesprek in gaat	1
Het onderwerp is belangrijk	1
Moeilijke onderwerpen zijn ook geschikt voor bijeenkomsten	1
Omdat we blijven herhalen	1

Onderwerp spreekt aan: nog niet helemaal in de vingers	1
Met het onderwerp kun je ook je kennis toetsen	1
Als je veel over ht onderwerp weet kan het zelfvertrouwen geven	1
Onderwerp spreekt me niet aan	1
Dit is een ondergeschoven kindje vandaar die blinde vlekken	1
Er zijn meningen over de standaarden	1
Discussie is afhankelijk van soort onderwerp	3
Alle onderwerpen geschikt voor groep	1
Onderwerp zonder duidelijke richtlijn	0
Uitermate geschikt om in de groep te bespreken	1
vooral als er veel ruimte in het onderwerp is	1
Veel ruimte in de standaard	1
veel onduidelijkheid waardoor meer subjectiviteit v. toepassing	2
Je moet doel goed voor ogen houden om niet te gaan kabbelen	1
Iedereen mag voor zichzelf referentiepunt bepalen	2
Onderwerp met duidelijke richtlijn	0
De standaard was heel ingewikkeld	2
Juist daardoor behoefte aan meer tools	1
Je weet dan beter hoe je het doet en wat je anders moet doen	1
Je kunt van elkaar leren in de manier waarop je ermee omgaat	1
Leerproces	0
Verschillende leerstijlen	0

Niet meteen normeren, eerst zoeken 'hoe zit dit dan?'	1
Er worden veel stappen overgeslagen	1
Je moet eerst onderzoeken wat je doet	1
Leeropbrengst deze bijeenkomst	0
Goed om het weer terug te halen	1
Het werkt samenvattend van wat er besproken iseen soort 'oh ja'	1
Vooral veel aan het denken gezet	1
Nadenken over hoe je het eigenlijk doet	1
door de leerpunten van anderen	1
Gevoeliger willen zijn voor bepaalde klachten	1
Minder vitamine d voorschrijven	2
Niet goed op veranderpunten doorgepakt	1
Er mist een handreiking tav wat je er mee kunt	1
Er zijn heel veel onbeantwoorde vragen	1
ook meteen leefstijladvies geven bij de uitleg	1
Bewustwording 'ik ga er op letten'	2
Maximale herhalingen in het HIS borgen	1
Leeropbrengst algemeen	0
Minder helder voor ogen dan andere deelnemer	1
Nabespreken met collega id praktijk: wat gaan we doen?	3
Uiteindelijk weing mee gedaan; niemand met die klachten gezien	1
Opbrengst van aios	1

Na bijeenkomst leergesprek gehad	0
door leergesprek is het meer gaan landen	2
Kon geen specifieke leerpunten noemen, het moest nog landen	1
Leervraag blijft eigenlijk onbeantwoord	1
wil niet bot overkomen, dus ik zeg het niet	1
Kwetsbaarheid en onzekerheid wordt geventileerd	0
Het leren boeit niet, ik blijf zelf uitzoeken	1
Hoeft niet altijd wat op te leveren	1
De groep zal het niet zeggen als het effect heel klein is	1
Het heeft geen zin om terug te komen op het actieplan	1
Openheid is nodig voor het leereffect	0
Achterste van je tong laten zien	1
Brengt denkproces op gang	2
Gamechangers	0
Genuanceerde leeropbrengst	1
Verbeterplannen doorvoeren	0
Als urgentie niet voor het voetlicht is, kans is dan klein om	1
Te weinig doen met de onbeantwoorde vragen	1
Niet maximale verwachten, maar haalbare	2
Het is veel te groot ding om door te voeren	1
Niet alle verbeterplannen worden doorgevoerd	1
Huisartsen zijn wandelende verbeterplannen	1

Cijfers	0
Hoor bij de cijfers zoveel mogelijk omdat ik het lastig vind	1
Aan de groep is te zien dat ze het cijfer gedeelt leuk vinden	1
De cijfers zeggen niet zo verschrikkelijk veel	1
Klopte niet	2
Groepsdynamiek	0
Sfeer	0
Ontspannen	1
Het gevoel dat je alles kan zeggen	1
Het is leuk	3
Er moet ruimte zijn voor enthousiasme en dat van elkaar voelen	1
Veiligheid	0
Het belangrijkste is dat ht echt veilig moet zijn	1
Intens om aangesproken te worden op voorbereiding	1
Niet erg vriendelijk	1
Het is geen veilige groep om dingen te zeggen	1
Het is intimiderend door aanwezigheid van opleiders	1
Beleefdheid hebben om naar een ander te luisteren	1
Beetje de draak steken hoort bij spelregels vd groep	1
Er is ruimte om met verrassingen te komen	1
Er is geen oordelende sfeer	1
Er wordt naar elkaars mening geluisterd	1

Het is een veilige groep	5
Er is respect onderling	2
Samen op een eerlijke en open manier naar kijken	1
Veiligheid ontstaat als je het een langere tijd met elkaar doet	4
Voelt je niet geremd om je mening te geven	4
Voorzichtig zijn in wat je zegt	1
Ik ga niet mijn nek uitsteken	2
Mijn mening zal wel fout zijn	1
Openheid	0
Ondanks de dynamiek in de groep is er openheid	1
Er is openheid	1
De groep vertrouwen om uitgebreid alles te ventileren	1
Openheid is nodig voor het leereffect	1
Achterste van je tong laten zien	1
Brengt denkprocos op gang, meer dan cijfers alleen kunnen doen	1
Iedereen is best open	1
Voelt je niet geremd om je mening te geven	1
Het moet wel leuk blijven	1
Humorvol	2
Er moeten wel gelachen worden	2
Er wordt veel gelachen	0
Het was een lacherig onderwerp: man-vrouwonderwerp	1

Het is een leuke groep om samen te leren	1
Omdat het een zinvolle activiteit is waarin we net meer leren	1
Dat is mijn grootste troef	1
Gewoon gezellig wat oplepelen	1
Het moet iets zijn waar mensen graag naar toe gaan	1
Rol andere groepsleden	0
Boetekleed aantrekken	5
Elkaars patroon herkennen	2
Rol moderator	0
Moderator is een beetje geweten van de groep	1
Iedereen in de groep kritisch maken	1
De mening van deelnemers uitlokken	2
zodat meer mensen wat gaan zeggen	1
Moderator duwde mij een bepaalde kant op	1
werkte averechts; toch mezelf afzijdig houden	1
Persoonlijkheid	0
Niet heel autoritair	1
Respectvol zijn	1
Heel erg open zijn	1
Moderator is nog voor leiding en organisatie	1
goed om te interrupteren	1
Niet overal druk om moeten maken, anders verlies je	1

Discussie openstellen	1
Checken of iedereen voorbereid heeft	1
Deelnemers aanspreken dat het als huiswerk was opgegeven	1
Bemoeizuchtig maar ook groepsgenoten hun ding willen laten doen	1
belangrijk dat groepsgenoten wat voorbereidein	1
Moderator zijn bij de gratie dat anderen ook voorbereiden	1
Structuur en proces in de gaten houden	2
Tijd goed benutten	1
Aansturen (veiligheid en inhoudelijk)	1
Houdt het proces in de gaten en brengt ook in	2
Gesprek terug brengen naar algemeen gedrag van de groepsleden	1
Dubbelrol moderator en groepslid	0
Moderator als onderdeel van de groep	1
Ervaringen delen zorgt voor meer openheid	1
Doet niet meer of minder mee dan de rest vd groep	1
Lekker meelullen	1
Het helpt niet als moderator ook ervaringen deelt	1
Dan blaf ik eventjes, maar daar ben ik heel zuinig mee	1
Verantwoordelijk dat de bijeenkomst niet mislukt	1
Niet er hard achteraan jagen op leerpunt	1
Kan niemand dwingen om achterste van hun tong te laten zien	1
probeert iedereen het woord te geven	2

Beschouwt eigen enthousiasme als gouden standaard	1
Als ik het leuk vind, vinden de collega's het ook leuk	1
Neutraal zijn, en oprecht nieuwsgierig	1
Partonen van deelnemers herkennen	2
Aparte rol aanmeten	1
Die moet je uit de tent lokken	1
Het goede voorbeeld geven	1
Moet ook bereid zijn om werkwijze aan te passen	2
Ontbrekende leerpunten vd rest als eigen leerpunt inbrengen	2
Zelf mijn nek het eerste uitsteken	2
Huisarts als moderator	1
Als huisarts ken je manier van werken	1
Niet kunnen ingrijpen omdat je als HA ook associatief denkt	1
Kan beter aanvoelen waar je moet kaderen	1
Moderator trekt groep met zich mee	4
Grapje draagt bij aan motivatie om terug te komen	2
Door enthousiast en niet belerend zijn	1
Moderator houdt je bij de les	1
Praktijk onderling	0
Samen een leerpunt opvatten, als praktijk	1
Afstemmen	0
Samen een leerpunt/actie aanpakken	1

Even overleggen wat er bij ons in de praktijk gebeurt	1
Samen verantwoorden hoe we hierin staan	2
Groep als geheel	0
De groep is redelijk neutraal	1
Huisartsen zijn altijd leuke mensen	0
Heel makkelijk om in een groep te zitten	1
Staan altijd open voor verbeteren en transparant naar zichzelf	1
Grootste deel is makkelijk zelfkritisch	1
Groepsinteractie	0
Tijd nodig om te voelen en iets te vinden om te delen	1
Het gevoel dat iedereen id groep zn vragen durft te stellen	2
Had meer willen zeggen, maar expert pakte dat niet goed op	1
Voelde niet vrij genoeg om daar meer over uit te leggen	1
Kritische HAen die op eigen manier naar de stof kijken en delen	1
Beetje draak te steken hoort bij de spelregels vd groep	1
De aios hoor je niet zo veel	2
aios: eigen mening minder van toegevoegde waarde	1
zit hard te pennen en weinig te reflecteren	1
Iedereen is even stil en aan ht nadenken	2
Het is helder wat de expert bedoelt en wat je moet doen	1
Een makke van expert is veel kennisdingen ophalen	1
De expert praat veel	2

Er worden veel vragen aan de expert gesteld	1
Er wordt te weinig gezegd	1
Cijfers zeggen weining over gedrag	1
Parameter staat te ver van wat er in de spreekkamer gebeurd	1
Vervelend om over de interpretatie te moeten discussieren	1
Onduidelijk of de groep luistert naar de vraag	1
Aanwezigheid van gasten werkt niet remmend	2
Disccusie volledig door elkaar heen	0
De discussie laten gaan en zelf uitvogelen hoe je het doet	1
Moeiljik te volgen, tijd nog om de cijfers te begrijpen	2
Grootste deel is makkelijk zelfkritisch	1
Verleidelijk om als groep hetzelfde gedrag te vertonen	1
Het zijn meemensen	1
Elkaar kennen	1
Op het gebied van kennis naar de moderator opkijken	1
Je bent meer open naar elkaar	1
Je vertrouwt elkaar	1
Alles wat iemand zegt kun je beter plaatsen	2
De deelnemers kennen elkaar goed	1
Moderator herkent patroon van deelnemers	1
Moderator kent expert	1
Moderator weet dat de expert een bepaalde toon heeft	1

Sommigen kennen elkaar heel goed	1
Groepssamenstelling is belangrijk	0
aios met minder ervaring vs. huisartsen met veel ervaring	0
Leerzaam omdat ervaren huisartsen hun ervaringen delen	1
Hun gefundeerde meningen horen is van toegevoegde waarde	1
aios: eigen mening minder v. toegevoegde waarde	2
Er zitten ook veel externe gasten bij	1
Aanwezigheid van gasten werkt niet remmend	1
Het is een leuke groep	2
Veiligheid	0
Heti s een veilige groep	1
De wijkgroep heeft zorgplicht, iemand buitensluiten kan niet	1
Eén aparte vogel kan, maar de niet de helft vd groep	1
Moet besproken worden of zo'n iemand erbij kan	2
Wat breng je mee?	0
Deelnemer let heel goed op en geeft veel	1
Praktische zaken	0
Inrichting bijeenkomst	0
Logistiek handiger om enquete/cijfer deel uit elkaar te trekken	1
Voorbereiding	0
Met de expert	0
Met het netwerk	0

Uitleggen waarom er voor bepaalde parameters gekozen is	2
Wat betekenen de cijfers	1
Hulp bij interpretatie van de cijfers	3
Voorbereiding deelnemers	0
voordeel van zo'n groep is dat je gaat voorbereiden	3
Je gaat er niet helemaal blanco in	1
voorbereiding kan je niet afdwingen	1
Realiteit is dat mensen niet altijd alles kunnen voorbereiden	1
De standaard is niet makkelijk om voor te bereiden	1
Eigenlijk moet iedereen de standaard voorbereiden	4
Iedereen die de standaard heeft gelezen moet het kunnen snappen	1
Voorbereiding moderatoren	1
Deelnemers verzoeken om enquete in te vullen/nadenken	1
Inventariseren welke onderwerpen leuk/relevant zijn	1
Van tevoren vragen wat voor hulp ze nodig hebben	1
Lastig balans te vinden in hulp aanbieden en volledig overnemen	3
Geen tijd om onderwerp voor te bereiden	1
Ontstaan van deze groep	0
Moderator is er ingerold	1
Half A4tje met onderwerpen voor de bijeenkomsten	1
Met de groep huisartsen oude Hagro	2
Stoppen van Hagro	1

Motivatie?	0
Grapjes maken draagt bij aan motivaitie om terug te komen	1
Deelnemers moeten het niet voor de moderator doen	2
In het algemeen	0
Leren	0
d.m.v. cijfers	0
Niet op harde normen spiegelen	0
d.m.v. meningsvorming	0
Groepsdynamiek	0
Sfeer	0
Rol andere groepsleden	0
Rol moderator	0
Groep als geheel	0
Cijfers	0
Leeropbrengst	0
