# Supplementary material for: Beyond Qualification: A Video-Stimulated Interview Study on What Group Discussion of Audit and Feedback Adds to Professional Development in General Practice
Source: Perspect Med Educ. 2026 Oct 12;15(1):1073–84. doi: 10.5334/pme.1954 (PMC13674528; doi:10.5334/pme.1954)
Supplement: Supplementary file 3 [file pme-15-1-1954-s3.pdf]

Codebook Deducative Analysis

Code System	Frequency
kwalificatie	21
klacht van patient als kwalificatie	1
verandering in praktijkvoering entameren	9
lastig	1
moeilijk door bureaucratie	1
inhoudelijke en procedurele kennis opdoen	9
voldoen aan eisen voor kwalificatie	3
nieuwe ontwikkelingen leren kennen	13
gaat met groep sneller dan alleen	3
leren van ervaren collega's	16
grote mond opzetten werkt niet	1
vakinhoudelijke sturing/expert	13
samen bepalen wat wij willen leren	3
blinde vlek wordt duidelijk	6
iedereen moet meedoen	4
subjectificatie	26
bewust worden van eigen handelen/normen en waarden	9
verantwoordelijkheid voor eigen patienten	2
eigen mening vormen	17

eigen handelen toetsen aan collega's	10
nieuwe invalshoeken/ideeën vormen	4
ik sta er toch iets anders in dan mijn collega’s	3
het individueel allemaal je eigen mening	18
reflecteren/eigen aanpak onder de loep nemen	16
verklaring waarom je niet met groep meedoet	10
zelf energie er in stoppen	10
Ik denk dat openheid wel nodig is voor het leereffect.	2
socialisatie	26
als solist extra belangrijk	2
van elkaar leren	6
persoonlijke ervaringen delen	7
elkaar leren kennen	6
professionale ervaringen delen	13
frustraties delen	2
goede voorbeelden zien	10
samen normen bepalen	9
vreemde eend includeren	6
veilige groep	12
groep niet te groot, niet te klein	1
voor jonge onervaren collega's lastig	3
lang bestaande onderlinge relaties	5

onderling respect	1
vrij voelen	1
gelijkgestemden	6
eerlijk/open	9
eigen mening naar voren te brengen	10
durft fouten toe te geven	2
niet oordelen	3
betrokkenheid	2
zelfde publiek/buurt	3
e hoopt dat anderen zeggen “oh, daar wil ik wel op inhaken”	1
de nieuwe generatie huisartsen includeren	2
aios sluit aan	4
groepsvorming	18
inbreng in groep	1
gezellig/plezier	12
als groep relevante relaties aangaan	4
moderator is deel van groep	8
duidelijke structuur	1
goede vooraf info	1
Dan kun je dus niet iemand van buitenaf zo makkelijk laten mode	1
onderlinge afstemming	8
RED	2

Laatste wat ik wil is geneeskunde vanuit eigen emotie bedrijven	1
Leereffect verbeteren door 2e keer langs te laten komen	1
MAGENTA	25
